# Effects of Low-Fish-Meal Diet Supplemented with Coenzyme Q10 on Growth Performance, Antioxidant Capacity, Intestinal Morphology, Immunity and Hypoxic Resistance of *Litopenaeus vannamei*

**DOI:** 10.3390/antiox12122042

**Published:** 2023-11-24

**Authors:** Yantao Liu, Zhenxiao Zhuang, Zhihong Liao, Rong Yao, Mengdie Chen, Hanlin Wei, Wei Zhao, Jin Niu

**Affiliations:** State Key Laboratory of Biocontrol, Guangdong Provincial Key Laboratory for Aquatic Economic Animals and Southern Marine Science and Engineering Guangdong Laboratory (Zhuhai), School of Life Sciences, Sun Yat-Sen University, Guangzhou 510275, China; liuyt38@mail2.sysu.edu.cn (Y.L.); zhuangzhx@mail3.sysu.edu.cn (Z.Z.); liaozhh25@mail2.sysu.edu.cn (Z.L.); yaor5@mail2.sysu.edu.cn (R.Y.); chenmd8@mail2.sysu.edu.cn (M.C.); weihlin@mail3.sysu.edu.cn (H.W.)

**Keywords:** white shrimp, coenzyme Q10, antioxidation property, immune response

## Abstract

The aim of this study was to evaluate the effects of a low-fish-meal diet supplemented with coenzyme Q10 on the growth, antioxidant capacity, immunity, intestinal health and hypoxic resistance of *Litopenaeus vannamei*. *L.vannamei* with an initial weight of 0.66 g were fed with the experimental diets for 56 days. Diets D1 (20% FM level) and D2–D7 (15% FM level), supplemented with 0%, 0.002%, 0.004%, 0.006%, 0.008% and 0.01% coenzyme Q10 were formulated. In terms of growth performance, the weight gain and specific growth rate in the D2 diet were significantly lower than those in the D1 diet (*p* < 0.05). The final body weight, weight gain and specific growth rate in the D2–D7 diets had an upward trend, and the condition factor in the D2–D7 diets was lower than those in the D1 diet (*p* < 0.05). There were no significant differences in the crude protein and crude lipid levels in the whole body among all diet treatments (*p* > 0.05). In terms of hepatopancreas antioxidant parameters, the D5 and D6 diets significantly promoted the total antioxidant capacity and total superoxide dismutase activity, and significantly decreased the malondialdehyde content (*p* < 0.05). The expression levels of *cat*, *mnsod* and *gpx* in shrimp fed with the D5 and D6 diets were significantly higher than those of shrimp fed with the D2 diet (*p* < 0.05). In addition, the mRNA level of *ProPO* was increased in the D4 and D5 diets, and *LZM* expression was increased in the D6 diet compared with the D1 diet (*p* < 0.05). The villus height of shrimp fed with diets supplemented with coenzyme Q10 was significantly increased (*p* < 0.05), and the intestinal thickness and submucosal thickness of shrimp fed with the D6 diet were the highest (*p* < 0.05). After acute hypoxia stress, lethal dose 50 time in the D3–D7 diets was significantly increased compared with the D1 and D2 diets (*p* < 0.05), and the highest value was found in the D4 diet (*p* < 0.05). After stress, the expression levels of TLR pathway-related genes (*Toll*, *Myd88*, *Pelle*, *TRAF6* and *Dorsal)* in the D4 and D6 diets were significantly increased compared with the D2 diet. In general, *Litopenaeus vannamei* fed with the D6 diet achieved the best growth, antioxidant capacity, immunity, and intestinal morphology among all low FM diets and D4–D6 diets improved hypoxic resistance.

## 1. Introduction

*Litopenaeus vannamei* is one of the most important crustaceans in aquaculture. Its aquaculture industry is developing rapidly because of its adaptability to wide temperature and salt, fast growth rate, and large meat yield. In 2021, *Litopenaeus vannamei* is the largest cultured shrimp species in China, with an area of 178,043 hectares and a yield of 1,273,632 tons [1].

Fish meal (FM) is a vital ingredient in aquatic feed and the main source of animal protein [2]. Plenty of studies have shown that *L. vannamei* has become one of the largest FM consumers [3,4]. Despite FM containing several beneficial amino acids, essential fatty acids, nucleotides, phospholipids, fats, minerals, and water-soluble vitamins, the price FM is continuously rising due to the rapid and influential expansions in the aquaculture sector [5,6,7,8]. Fluctuations in the supply, price, and quality of FM have become impending risks [9]. In recent years, there have been significant developments in research into FM replacement in aquaculture, but low-FM feed has several nutritional drawbacks [10,11,12,13], such as a worse growth performance, antioxidant capacity, immunity performance, etc. [14,15,16]. Sustainable alternative protein sources with lower market prices have partially or completely replaced FM for aquafeed. Nowadays, FM can be replaced by several sustainable protein sources like plant protein sources and animal protein sources [17]. But, compared with FM, these alternative protein sources have a number of disadvantages. Compared to plant protein, animal protein sources, such as meat and bone meal (MBM), blood meal (BM), insect meal, and poultry by-product meal (PBM), have the advantages of a higher protein and lipid content, a more suitable amino acid composition, and better palatability [9]. But some animal sources also have deficiencies or excesses in essential amino acids [18,19] (EAAs). The fluctuation of quality of animal protein sources depends on variability in raw material composition, processing and quality [20]. Although plant protein sources such as soybean meal (SBM), peanut meal (PM), and corn gluten meal (CGM) contain a high fiber content, they have a low protein content, a lack of one or more EAAs, low palatability and several antinutritional factors (ANFs), such as protease inhibitors, tannins, oligosaccharides and phytates, which reduce nutrient availability and digestibility of feed; however, they also have lower market prices, a large quantity in production and a stable quality, which make them suitable for FM replacement and reducing the cost of feeds [21,22,23,24]. To reduce the drawbacks, different functional additives are supplied in diets to maintain feed palatability, promote growth performance, or enhance immunity, aspects that are probably directly impacted by using FM replacement feeds [25,26,27,28,29,30,31].

Coenzyme Q10, also known as ubiquinone, is a hydrophobic molecule [32]. It is a naturally occurring compound whose properties are similar to vitamins (vitamin Q) [33,34]. As a cofactor in the mitochondrial electron transport chain (respiratory chain), coenzyme Q10 plays an important role in cellular bioenergetics and is critical for the production of ATP. Coenzyme Q10 acts as a mobile oxidant-reducing agent, shuttling electrons and protons in the electron transport chain. The redox function of coenzyme Q10 goes far beyond its form in mitochondria [35,36]. In addition, coenzyme Q10, as a reduced form of hydroquinone, serves as a potent antioxidant in mitochondria and lipid membranes, capable of recovering and regenerating other antioxidants, such as tocopherol and ascorbic acid [37]. Furthermore, coenzyme Q10 can also be involved in cell signaling and gene expression as a membrane stabilizer [38].

Coenzyme Q10 has been extensively studied for its strong antioxidant properties in humans and some animals [39,40,41]. Substantial studies show that coenzyme Q10 has enormous potential in the treatment of diseases, such as diabetes, obesity, muscular dystrophy, cancer, heart disease, oral wounds, Alzheimer’s disease, Parkinson’s disease, aging and other diseases [41,42,43,44,45,46,47,48]. In addition, coenzyme Q10 has been proven to have anti-inflammatory properties in vitro [49]. Coenzyme Q10 can effectively inhibit the oxidation of lipids and proteins, as well as DNA damage [50]. Due to its potential superior function in treating diseases and its advantages of easy synthesis by biological or chemical methods, coenzyme Q10 is a special material with great demand in the market. In recent years, coenzyme Q10 has attracted widespread attention as a feed additive for aquatic economic animals.

The aim of this study is to investigate the effect of supplementing coenzyme Q10 in low-fish-meal feed on the growth immunity, antioxidant status, intestinal morphology and acute hypoxic resistance of *L. vannamei*. The findings of this study will provide data support and a theoretical basis for the development of low-FM diets for *L. vannamei*.

## 2. Material and Methods

### 2.1. Ethics Approval

All experiments were conducted in accordance with the recommendations in the Guide for the Care and Use of Laboratory Animals of the National Institutes of Health (NIH). The study protocol and all experimental procedures were approved by the Institution Animal Care and Use Committee, Sun Yat-Sen University (approval code: SYSU-IACUC-2022-B0159, approval date: 4 March 2020).

### 2.2. Diets Design and Preparation

In this study, the shrimp were randomly divided into seven experimental groups, presented in Table 1. Among them, D1 contains 20% FM, and six other diets were prepared with 15% FM (25% of the FM replaced with corn gluten meal). Coenzyme Q10 in proportions of 0, 0.002, 0.004, 0.006, 0.008 and 0.01% were supplemented in D2, D3, D4, D5, D6, and D7 diets, respectively (0, 2, 4, 6, 8, 10 mg/kg of coenzyme Q10). The method of diet preparation was similar to that described by Liao et al. [51]. Dry ingredients were ground, weighed, combined and mixed well to homogeneity in a Hobart-type mixer (A-200T Mixer Bench Model unit, Resell Food Equipment Ltd., Ottawa, ON, Canada). Then, soy oil, fish oil and soybean lecithin were added and thoroughly mixed for 5 min. Water (40% dry ingredients mixture) was added and mixed well for 10 min. The 1.2 mm diameter pellets were extruded using a pelletizer (Institute of Chemical Engineering, South China University of Technology, Guangzhou, China), and heated in an electric oven at 80 °C for 1.5 h. All the diets were air-dried to 10% moisture and stored at −20 °C until used.

### 2.3. Rearing Conditions and Sampling

An 8-week feeding experiment with *L. vannamei* was conducted in the Sanya experimental base of South China Sea Fisheries Research Institute (Sanya, China). Before the experiment, all the shrimp were fed with commercial feed (crude protein content is 42%, crude fat content is 5%) and acclimated for 6 weeks. At the beginning of the experiment, 840 healthy shrimp with an average weight of 0.67 g ± 0.017 g were randomly distributed into 28 fiberglass tanks (400 L, 0.7 m^2^ bottom, and 4 tanks per diet) at a density of 30 shrimp in each tank. All shrimp were fed to apparent satiation three times daily at 8:00, 15:00, and 22:00. During the period of the experiment, the conditions were as follows: PH: 7.7–8.2; water temperature: 29.2–30.0 °C; dissolved oxygen > 6.5 mg/L; salinity: 35‰. The normal natural light cycle was maintained throughout the experiment.

After the 8-week feeding experiment, all the shrimp were fasted for 24 h before sampling. Then, all the live shrimp in each tank were counted and weighed. A total of 5~8 shrimp in each tank were collected to analyze the proximate compositions of the whole shrimp body. The hepatopancreas of 4 shrimp were collected to measure enzyme activity and gene expression under aseptic conditions, then immediately placed in liquid nitrogen and stored at −80 °C. Two pieces of midgut tissue were obtained after dissection from 2 shrimp, which were fixed in 4% paraformaldehyde solution for 24 h, then transferred to 70% ethanol solution for fixation and stored at room temperature for subsequent HE staining section analysis.

### 2.4. Acute Hypoxic Stress Experiment

After sample collection, 10 shrimp were randomly selected from each tank and put into the original tank to calm down the stimulation for the subsequent acute low-dissolved-oxygen stress experiment. In the experiment, sample bags with unified specification were filled with the same amount of seawater, and the live shrimp in each tank were transferred to the corresponding sample bags and sealed. The number and time of dead shrimp in each group were recorded until half of them died, and the response time when half of them died was counted. Before the experiment, the dissolved oxygen in water was detected by the dissolved oxygen meter, and the dissolved oxygen before stress was approximately about 6 mg/L. The salinity was 35‰, and ammonia nitrogen level was not more than 0.1 mg/L.

### 2.5. Analysis of the Whole Body Proximate Composition

Moisture, crude lipid and crude protein in the diets were determined using standard methods (AOAC, 1995). The whole shrimp body was dried in the oven at 105 °C to constant weight. Meanwhile, moisture was determined. Crude protein was measured by the Kjeldahl method after acid digestion using an Auto Kjeldahl System (1030-Auto-analyzer; Tecator, Hoganos, Sweden). Crude lipid was determined by the ether-extraction method using a Soxtec System HT (Soxtec System HT6; Tecator, Sweden).

### 2.6. Intestinal Morphology Analysis

Two matching segments of midgut tissue were obtained after dissection from 2 shrimp and placed into 4% paraformaldehyde solution at room temperature for 24 h immediately, then were fixed in 70% ethanol as follows: The samples were dehydrated by a series of different graduation of ethanol and then embedded in paraffin. The paraffin-embedded blocks were cut into sections with a thickness of about 3–5 μm before being stained by using hematoxylin/eosin. The stained sections were observed and photographed with a Nikon upright microscope (Eclipse NI-E, Japan). The average intestinal villus height and submucosal thickness were randomly determined within 5 different fields and four replicates for each group.

### 2.7. Antioxidant Capacity Examination

About 0.6 g of the hepatopancreas tissue of *Litopenaeus vannamei* was homogenized with cold PBS (PH:7.4) according to the ratio of the sample weight: PBS volume (w:V) =1:9. Then, the homogenate was centrifuged (4000 rpm, 4 °C) for 20 min and the supernatant was used for determination. Total superoxide dismutase (T-SOD), total antioxidant capacity (T-AOC) and malondialdehyde (MDA) activities were analyzed using commercial kits (T-SOD, A001-1-2; T-AOC, A015-3-1; Nanjing Jiancheng Bioengical Institute, Nanjing, China; MDA, S0131S; Beyotime Biotechnology Institute, Shanghai, China).

### 2.8. Quantitative Real-Time PCR Analysis

All gene expression levels in this study were detected using real-time quantitative PCR. Total hepatopancreas RNA was extracted using an RNAeasy™ Animal Long RNA Isolation Kit (Beyotime Biotechnology Institute, Shanghai, China) following the manufacturer’s protocol. After the extraction, the concentration and quality of total RNA were determined using an ultrafine spectrophotometer Nanodrop-2000 (Thero Scientific, Waltham, MA, USA, A260: A280 nm). After this, the genomic DNA was removed by using an Evo M-MLV RT Kit with gDNA Clean for qPCR Ⅱ (Accurate Biology, Changsha, China). The reaction condition was 42 °C for 2 min, and the DNA was stored at 4 °C after the reaction. Then, complementary DNA (cDNA) was synthesized using reverse transcription. The program was performed following the reaction conditions: 37 °C for 15 min, 85 °C for 5 s; it was then diluted to 200 μL with DEPC water and stored at −20 °C.

The qRT-PCR for the genes was performed on a LightCyler480 (Roche Applied Science, Basel, Switzerland). All the qRT-PCR primers for targeted genes, designed by SnapGene based on the published nucleotide sequences, are shown in Table 2. For each sample, three technical replications were run. The qRT-PCR reaction steps were as follows: 95 °C for 10 min, then 40 cycles subsequently (95 °C for 5 s, 60 °C for 30 s, 72 °C for 30 s), and finally cooled down at 4°C. Normalization of the gene expression was performed by a reference gene (β-actin), and expression levels were quantified using the 2^−ΔΔCt^ method.

### 2.9. Calculations and Statistical Analysis

The parameters were calculated as follows:

Initial body weight (IBW, g) = initial body wet weight (g);

Final body weight (FBW, g) = final body wet weight (g);

Weight gain rate (WG, %) = 100 × (final body weight − initial body weight)/initial body weight;

Specific growth rate (SGR, % day^−1^) = 100 × (Ln (final mean weight) − Ln (initial mean weight))/number of days;

Feed conversion ratio (FCR) = feed consumed (g, dry weight)/weight gain (g, wet weight);

Condition factor (CF, g/cm^3^) = 100 × (body weight, g)/(body length, cm)^3^;

Survival rate (%) = 100 × (final number of shrimp)/(initial number of shrimp).

All the data were presented as the means with SEM. Data from each treatment were subjected to one-way analysis of variance (ANOVA) and Duncan’s multiple tests. SPSS 22.0 statistical software was used to analysis the results. *p* < 0.05 was considered to indicate a statistically significant difference. A non-parametric Kruskal–Wallis test was applied when data did not have a homogeneous variance, followed by all pairwise multiple comparisons if there were significant differences (*p* < 0.05).

## 3. Results

### 3.1. Growth Performance and Feed Utilization

The growth performance and feed utilization of *L. vannamei* were shown in Table 3. After an eight-week feeding trial, the shrimp in all diets survived well (>85%), and there were no significant differences among all diets (*p* > 0.05). WG and SGR were influenced by dietary coenzyme Q10 levels. The WG and SGR of the shrimp fed with the D2 diet were significantly lower than the D1 group (*p* < 0.05). However, there were no significant differences between those fed with the D3, D4, D5, D6, and D7 diets and the D1 diet (*p* > 0.05). Shrimp fed with the D3, D4, D5, D6, and D7 diets had gradually increased FBW, WG and SGR with a rise in the coenzyme Q10 level. There were no significant differences in the FBW, FCR and Survival among all groups (*p* > 0.05). In addition, the groups fed with a lower FM level had a lower CF than D1 (*p* < 0.05), while there was no difference among the shrimp fed from diets D2 to D7 (*p* > 0.05).

### 3.2. Whole-Body Proximate Composition

The moisture, crude protein and crude lipid levels of the whole body are presented in Table 4. The moisture parameter showed significant differences among all groups (Table 4). D2, D3, D6 and D7 had higher moisture values than D1, D4 and D5 (*p* < 0.05). Moreover, the moisture values of shrimp fed with the D3, D6, D7 diets were higher than the D2 diet (*p* < 0.05). However, the crude protein and crude lipid of shrimp were not influenced by the FM level or coenzyme Q10 level (*p* > 0.05).

### 3.3. Hepatopancreatic Antioxidant Enzyme Analysis

**Figure 1:**
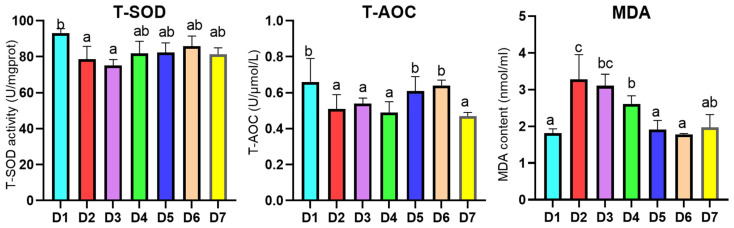
Effect of different diets on antioxidant capability in hepatopancreas of *L. vannamei*. Left: T-SOD, total superoxide dismutase; middle: T-AOC, total antioxidant capacity; right: MDA, malondialdehyde. The results are expressed as mean and error bar (SEM); 4 replications in each group (n = 4). a,b,c, shown above the error bars indicate a significant difference among groups; different letters displayed on top indicated significant difference (*p* < 0.05); same letters mean no significant difference (*p* > 0.05).

The results of the hepatopancreatic antioxidant enzyme activity of *L. vannamei* fed with different diets are shown in Figure 1. Results indicated that the T-AOC values of *L. vannamei* fed with the D2–D4 diets and the D7 diet were significantly lower than those fed the D1 diet (*p* < 0.05), but no significant differences among the D1, D5 and D6 diets (*p* > 0.05) were found. The T-SOD activity of shrimp fed with the D2 and D3 diets were remarkably lower than the D1 group (*p* < 0.05); on the contrary, no remarkable differences were found compared to the groups with a coenzyme Q10 level higher than 0.002% and the D1 group (*p* > 0.05). Analogously, the malondialdehyde (MDA) content of the shrimp fed with the D2 diet was the highest among all groups (*p* < 0.05), while those of the D5 and D6 diets were observably lower than D2–D4 diets (*p* < 0.05), and were no significant differences compared to the D1 diet (*p* > 0.05). Summarily, a certain extent of dietary coenzyme Q10 supplement can increase T-AOC and decrease MDA to improve the antioxidant capability of *L. vannamei*, but cannot affect the T-SOD activity of *L. vannamei*. Moreover, to a certain extent, *L. vannamei* fed a diet containing a low FM level (15%) with a coenzyme Q10 supplement can achieve the antioxidant capabilities of the shrimp fed with a high FM level (20%).

### 3.4. Relative Expression of Antioxidant and Immune Genes in Hepatopancreas

**Figure 2:**
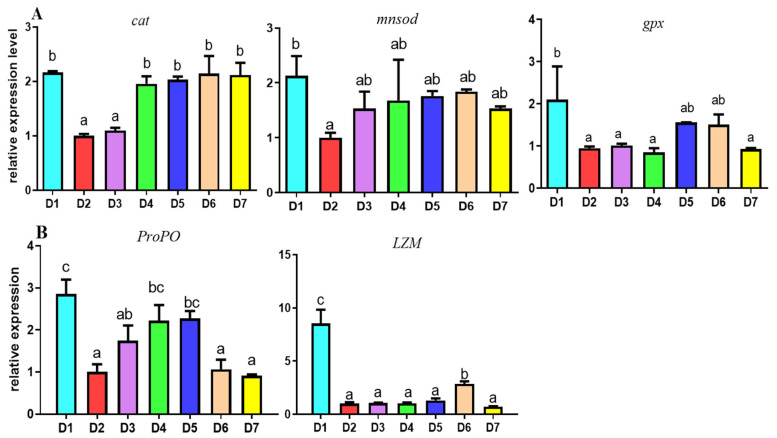
The relative antioxidant and immune-related gene expression levels in hepatopancreas of *L. vannamei* fed with different diets. (**A**) CAT, MnSOD, GPX, (**B**) ProPO, LZM genes of mRNA relative expression level, respectively. Abbreviation: CAT, Catalase; MnSOD, Manganese superoxide dismutase; GPX, Glutathione peroxidase; ProPO, Prophenoloxidase; LZM, Lysozyme. The results are expressed as mean and error bar (SEM); 4 replications in each group (n = 4); a,b,c, shown above the error bars indicate significant differences among groups; different letters displayed on top indicated significant difference (*p* < 0.05); same letters mean no significant difference (*p* > 0.05).

The results of the relative expression of antioxidant and immune genes in the hepatopancreas of *L. vannamei* were shown in Figure 2. The expressions of antioxidant genes are shown in Figure 2A. There were no significant differences among the shrimp fed with the D1 and D4–D7 diets in the *cat* mRNA expression level (*p* > 0.05), but the expression level in the D2 and D3 groups were significantly lower than the D1 group (*p* < 0.05). The results of the relative expression of *mnsod* mRNA indicated that it was distinctly lower in the D2 group compared with the D1 group (*p* < 0.05). In addition, the relative expression of *gpx* gene mRNA in D1 group was markedly higher than that in D2, D3, D4 and D7 groups (*p* < 0.05); however, there was no significant difference between the D1 group and the D5 and D6 groups (*p* > 0.05).

The expressions of immune genes in the hepatopancreas are shown in Figure 2B. The results indicated that the expression levels of *ProPO* mRNA were significantly higher in the D4 and D5 groups than the D2, D6 and D7 groups (*p* < 0.05), especially it was highest in the D1 group (*p* < 0.05); there were no significant differences between the D1, D4 and D5 groups. Moreover, the expression level of the *LZM* gene was highest in D1 group, and it was markedly higher than that in the D2–D7 groups (*p* < 0.05), but among the low-FM groups, it was highest in the D6 group (*p* < 0.05).

### 3.5. Intestinal Morphology Measurement

**Figure 3:**
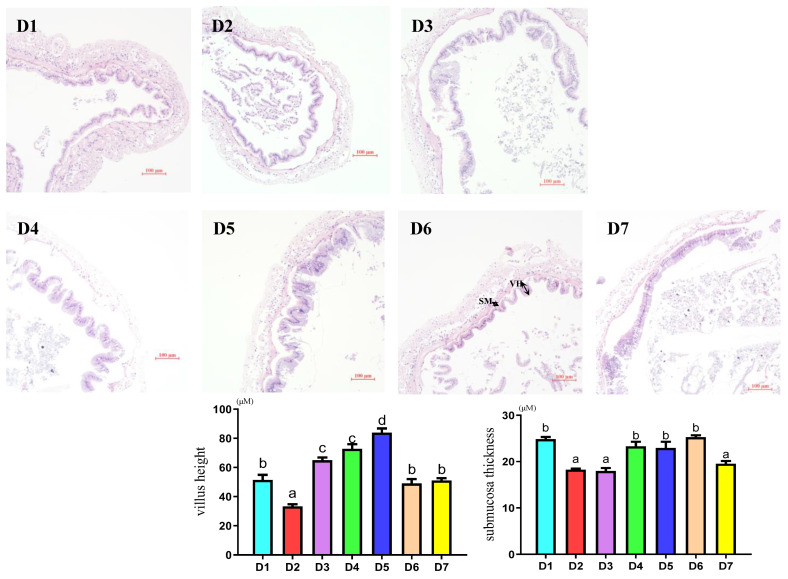
Effects of different supplemental levels of coenzyme Q10 on intestinal morphology of *L. vannamei.* Top: intestinal morphology; scale bar, 100 μm, original magnification ×10; VH: villus height; SM: submucosa thickness. Below: The results of villus height and submucosa thickness measurement. The average intestinal villus height and submucosal thickness were randomly determined within 5 different fields and four replicates for each group. The results were expressed as mean and error vertical bars (SEM); 4 replications in each group (n = 4). a,b,c,d shown above the error bars indicate significant differences among groups; different letters displayed on top indicated significant differences (*p* < 0.05); same letters mean no significant difference (*p* > 0.05).

The intestinal morphology measurements of *L. vannamei* from each group are shown in Figure 3. The intestinal villus height and submucosal thickness of *L. vannamei* from each group were determined. According to the intestinal morphology of *L. vannamei* in each group, shown by intestinal HE staining sections (bottom of Figure 3), neither the FM level nor coenzyme Q10 addition level caused pathological changes in the *L. vannamei* intestinal morphology. Statistically speaking, the intestinal villus height and submucosal thickness showed significant differences among all treatment groups. The intestinal villi heights of groups D3–D7 were significantly higher than that of group D2 (*p* < 0.05), and the intestinal villi height of groups D3–D5 was significantly higher than that of group D1 (*p* < 0.05). There was no significant difference between groups D6 and D7 and group D1 (*p* > 0.05). The submucosal thickness in the D4, D5 and D6 groups was significantly higher than that in the D2, D3 and D7 groups (*p* < 0.05), but there was no significant difference in the submucosal thickness between the D4, D5 and D6 groups and the D1 group (*p* > 0.05).

### 3.6. Acute Hypoxia Stress

**Figure 4:**
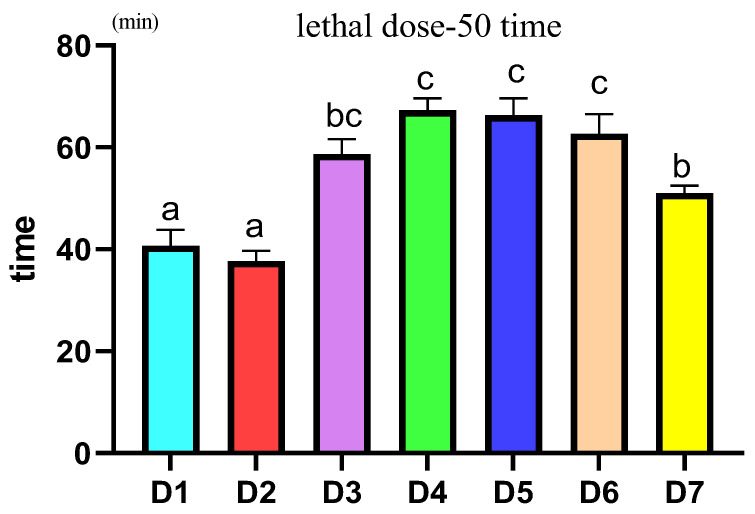
Effects of different supplemental levels of coenzyme Q10 on the lethal dose 50 time of *L. vannamei* under acute hypoxia stress. The results are expressed as the mean and error vertical bar (SEM); 4 replications in each group (n = 4). a,b,c, shown above the error bars indicate significant differences among groups; different letters displayed on top indicated significant difference (*p* < 0.05); same letters mean no significant difference (*p* > 0.05).

Figure 4 shows that the death time of half of *L. vannamei* under hypoxia stress was calculated between different treatment groups. According to the statistics in the figure, the median lethal time in the D3–D7 groups was significantly higher than that in the D1 and D2 groups (*p* < 0.05). Among all the treatment groups, the D4–D6 group had the longest half-death time, which was significantly higher than that of the D7 group (*p* < 0.05).

**Figure 5:**
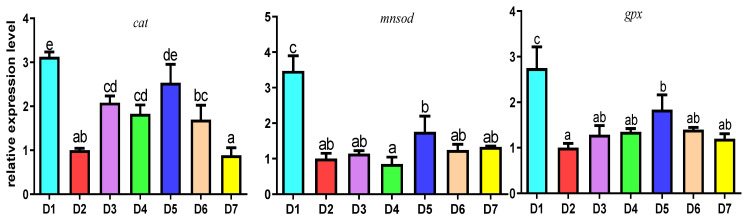
Effects of different supplemental levels of coenzyme Q10 on the expression of hepatopancreatic antioxidant-related genes of *L. vannamei* under acute hypoxia stress. The results are expressed as the mean and error vertical bar (SEM); 4 replications in each group (n = 4). a,b,c,d,e shown above the error bars indicate significant differences among groups; different letters displayed on top indicate significant differences (*p* < 0.05); same letters mean no significant difference (*p* > 0.05).

The results of antioxidant-related gene expression are presented in Figure 5. The *mnsod*, *cat* and *gpx* genes’ relative expression were significantly upregulated after diets supplemented with coenzyme Q10. For the result of *cat*, those of D3–D6 were markedly higher than that of D2 (*p* < 0.05); in particular, the D5 group was the highest among them, with no significant differences compared with D1 (*p* > 0.05). Similarly, the results of *mnsod* and *gpx* gene relative expression showed that D5 was the highest among D2–D7 (*p* < 0.05). The expressions of these antioxidant-related genes were boosted after proper supplementation with coenzyme Q10.

**Figure 6:**
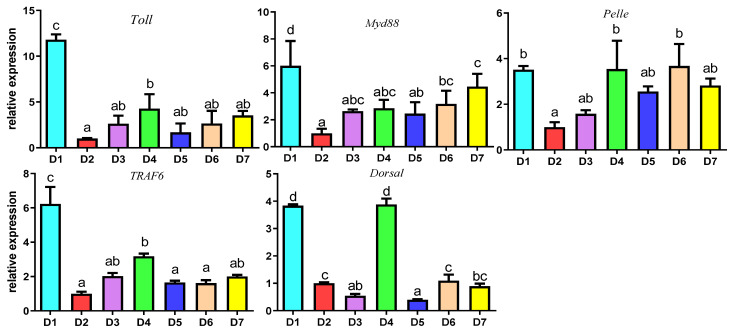
Effects of different supplemental levels of coenzyme Q10 on the expression of hepatopancreatic immune-related genes (Toll-like receptor signaling pathway) of *L. vannamei* under acute hypoxia stress. The results are expressed as the mean and error vertical bar (SEM); 4 replications in each group (n = 4). a,b,c,d shown above the error bars indicate significant differences among groups; different letters displayed on top indicated significant differences (*p* < 0.05); same letters mean no significant difference (*p* > 0.05).

Figure 6 showed the partial immune-related gene expression of *Litopenaeus vannamei* under acute hypoxia stress in different treatment groups. The mRNA relative expression levels of a Toll-like receptor signaling pathway-related genes (*Toll*, *Myd88*, *Pelle*, *TRAF6* and *Dorsal*) in the D2–D7 group were significantly lower than those in the D1 group (*p* < 0.05). In the D2–D7 groups, with a 15% fish meal level, the *Toll* expression level in the D4 group was significantly higher than that in the D2 group (*p* < 0.05), but there was no significant difference between the D4 group and the other 15% fish meal groups (D3, D5, D6 and D7) (*p* > 0.05). The expression levels of the *Myd88* gene in the D6 and D7 groups were significantly higher than that in the D2 group (*p* < 0.05), but there were no significant differences between the D2-D5 groups (*p* > 0.05). The *Pelle* gene expression level in the D4 and D6 groups was significantly higher than that in the D2 group (*p* < 0.05), but there were no significant differences between the D4, D5 and D7 groups and the D2 group (*p* > 0.05). The expression level of the *TRAF6* gene in the D4 group was significantly higher than that in the D2, D5 and D6 groups (*p* < 0.05), but there was no significant difference between the D4, D3 and D7 groups (*p* > 0.05). Interestingly, the *Dorsal* expression level in D4 was highest among D2–D7 (*p* < 0.05) and no remarkable discrepancy compared with D1 (*p* > 0.05).

## 4. Discussion

At present, FM is still the main source of protein in aquatic animal feed, but its price is increasing due to environmental impacts and overfishing [52,53]. Currently, FM levels in aquatic feed are usually reduced by substituting it with other protein sources. Diets with low fish meal levels, in particular, plant-based proteins (such as SBM, PM, CGM, dephenolized cottonseed protein, etc. [54,55,56,57]) are often less effective than high-fish-meal diets due to their high fiber content, low protein content, inappropriate amino acid composition, low palatability and the antinutritional factors of plant protein sources, which reduce the feed intake, nutrient availability and digestibility of feed, and influence the growth, antioxidant capacity and immunity of the shrimp [6,10,11,12,13]. In this study, CGM was used as an FM replacement and had a high content of non-soluble carbohydrates, which were hard for digestion and utilization and had adverse impacts on the shrimp [36,58]. In recent years, there have been an increasing number of studies focusing on low-FM feed for aquatic animals. In some cases, low-FM feed hindered the growth performances of aquatic animals such as turbot, rainbow trout, Atlantic Salmon, Nile tilapia, gilthead sea bream, *largemouth bass*, *L. vannamei*, black tiger shrimp, *Paralichthys olivaceus*, etc. [11,59,60,61,62,63,64,65,66]. A low-FM diet supplemented with functional additives to alleviate the growth retardation of aquatic animals has become a hot research objective in aquaculture. Coenzyme Q10, an inexpensive, obtainable, strong antioxidant dietary supplement, has been proven to yield a broad range of advantageous impacts [67]. In this study, the addition of coenzyme Q10 also produced a good effect on *L. vannamei*. In terms of growth performance, weight gain rate (WG) and specific growth rate (SGR) increased with the addition of coenzyme Q10. Similarly, two studies by Basuini et al. indicated that the addition of coenzyme Q10 increased the growth, in terms of the WG and SGR, of Nile tilapia, which might be caused by the enhancement of the feed intake (FI) and the feed efficiency ratio (FER) with the dietary coenzyme Q10 supplement [68,69]. The findings of this study suggest that low-FM diets impair shrimp growth compared with high-FM diets, but dietary coenzyme Q10 supplementation can reverse the growth retardation caused by low-fish-meal diets. By adding coenzyme Q10, the results of intestinal villus height were enhanced compared to shrimp in the no-coenzyme-Q10 groups, which possibly promoted feed utilization, and this might be one of the reasons for the boosted growth performance.

Increased ROS (reactive oxygen species) levels cause oxidative damage to many important cellular macromolecules (lipids, proteins, carbohydrates, and nucleotides) [70]. As antioxidant enzymes in *L. vannamei*, SOD (superoxide dismutase), CAT (catalase) and GPX (glutathione peroxidase) can remove excessive ROS in vivo. They exist in approximately all aquatic animals and are normally used to assess the antioxidant capacity of organisms [71,72,73]. SOD is a cytosolic enzyme that can be detoxified using a superoxide radical (O^2−^) converted to hydrogen peroxide and oxygen [74]. CAT (catalase) and GPX (glutathione peroxidase) can convert H_2_O_2_ into H_2_O [75,76]. The findings of this study showed that added coenzyme Q10 slightly increased the relative expression level of *MnSOD*, the same as the result of T-SOD activity. Moreover, the expression level of *GPX* was mildly increased when supplemented with 6 and 8 mg/kg of coenzyme Q10. Similarly, *CAT* expression was upregulated with coenzyme Q10 by higher than 4 mg/kg. The T-AOC (total antioxidant capacity) and MDA (malondialdehyde) are both indicators of the antioxidant capacity and oxidant status of *L. vannamei*. T-AOC directly reflects antioxidant capacity overall, while MDA, as the end product of lipid peroxidation, is an important indicator of cell-membrane oxidative damage [77]. In the present study, it was observed that the shrimp fed with a 20% FM diet had a higher T-AOC and a lower MDA than those shrimp fed with a 15% FM diet, but the low-FM diets supplemented with 6 and 8 mg/kg coenzyme Q10 reversed the antioxidant inhibition caused by the FM-meal diets. When coenzyme Q10 was added to grouper sperm frozen medium [78], it was observed that oxidative stress was reduced, which significantly improved sperm motility and the fertilization rate. Homoplastically, in a previous study on *L. vannamei*, the T-AOC activity was increased and the *SOD* expression level was promoted by adding coenzyme Q10 under a low salt stress [79]. The results indicated that the antioxidant system of *L. vannamei* can be promoted by adding coenzyme Q10. Therefore, in the present study, it was found that coenzyme Q10 could improve the nutritional physiology of *L. vannamei*, which might be related to the strong antioxidant capacity of coenzyme Q10 alleviating the oxidative stress of the organism. In the human body, coenzyme Q10 has been used as an added supplement for the treatment of various diseases, and some of the help that coenzyme Q10 gives to human diseases is based on its strong antioxidant ability to clean up lipid peroxidation and enhance mitochondrial respiration [80].

Coenzyme Q10 also possesses the potential ability to activate the immune system. There is considerable evidence that coenzyme Q10 may influence immunity via different mechanisms, such as its effects on pro-inflammatory markers, mitochondria, lysosomes and peroxisomes [81]. For *L. vannamei*, the prophenoloxidase system (proPO-system) and lysozyme are the keys of the nonspecific immune system. Prophenoloxidase activation plays an important role as a recognition system through blood cell attraction and the induction of phagocytosis, melanization, cytotoxic reactant production, particle encapsulation and the formation of nodules and capsules [82]. ProPO is a crucial enzyme in the prophenoloxidase system, which transfers to catalytically activated phenoloxidase (PO). Lysozyme lyses bacteria by attacking the peptidoglycan of the bacterial cell wall [83]. Results have showed that a dietary supplement of 4 and 6 mg/kg of coenzyme Q10 enhanced the relative expression level of *ProPO*, and 8 mg/kg of coenzyme Q10 upregulated lysozyme expression, which indicates the positive correlation between coenzyme Q10 and innate immunity. Coenzyme Q10 helps to maintain the acidic environment of the lysosomal lumen and also maintain the structural integrity of the peroxisome membrane from free-radical-induced oxidative damage, which may be one of the reasons why coenzyme Q10 enhances the innate immunity of *L. vannamei* [84,85].

Hypoxia is the main limiting factor in the crustacean aquaculture industry [78]. Both acute and chronic hypoxia stress can damage tissues in crustaceans [86,87]. The results indicated that coenzyme Q10 supplementation prolonged the semi-lethal time caused by acute hypoxic stress, which may be attributed to an improved health status and immunity. One of the deleterious consequences of hypoxia stress is the induction of ROS generation in aquatic animals [88]. Hypoxia increases ROS by transferring electrons from the ubisemiquinone to molecular oxygen at the Qo site of complex III of the mitochondrial electron transport chain [89]. Previous result have showed that hypoxia and subsequent reoxygenation induced high level of ROS in the hepatopancreas of *L.vannamei* [90]. In a study in Nile tilapia [91], the addition of coenzyme Q10 alleviated the oxidative stress caused by nickel to the brain, restored the activity of acetylcholinesterase, and alleviated the nerve damage caused by nickel to Nile tilapia. The results of this study showed that a proper coenzyme Q10 supplement enhanced the animals antioxidant capacity, and this will help to clarify the excess ROS that are being generated after hypoxia stress.

Hypoxia is known to hinder the nonspecific immunity system of crustaceans, which increases their susceptibility to pathogenic bacteria [92,93]. Hypoxia has been reported to reduce hemocyte counts and phagocytic activity, as well as reduce the bacteriolytic and antibacterial activity in the hemolymph of the southern king crab Lithodes santolla, giant freshwater prawn *Macrobrachium rosenbergii*, and white shrimp *L. vannamei* [78]. Hypoxia affects the expression of immune-related genes, but little is known about the mechanisms involved. The gills and hepatopancreas are the most sensitive organs in crustaceans and are susceptible to oxidative stress. In this study, the Toll-like receptor signaling pathway-related genes (*Toll*, *Myd88*, *Pelle*, *TRAF6* and *Dorsal*) were investigated after acute hypoxia stress. The Toll-like receptor signaling pathway plays a vital role in the innate immunity system, where it controls and clears invading pathogens [94,95]. Toll is a major pattern-recognition receptor of *L. vannamei*. It recruits the adaptor MyD88 through its own TIR domain, which then binds to the protein kinase Pelle to construct a complex that recruits other modulators. Phosphorylated Pelle induces the phosphorylation of tumor necrosis factor receptor-associated factor 6 (TRAF6), thereby inducing an immune response [94]. Dorsal, which belongs to the class II NF-κB family [96], is the critical regulator of the Toll-like receptor signaling pathway and is responsible for DNA binding, dimerization and interaction with the regulator proteins for the regulation of the expression of antimicrobial peptides [97]. The results of this study have shown that 4 mg/kg coenzyme Q10 supplementation enhanced the immunomodulatory effect of the Toll-like receptor signaling pathway after acute hypoxia stress, indicating that coenzyme Q10, which may help to enhance the animals antioxidant capability to fight oxidants that are generated after hypoxia stress, can help to improve the innate immune capacity of *L. vannamei*.

## 5. Conclusions

In this study, the results showed that dietary supplementation with an appropriate amount of coenzyme Q10 could enhance the nutritional physiological status of *Litopenaeus vannamei* fed on low-FM feed. In general, the optimum addition level of coenzyme Q10 was 0.008% for growth performance, antioxidant capacity, intestinal development, and immune performance. Dietary coenzyme Q10 supplementation in the amount of 0.004–0.008% improved the anti-hypoxic ability of *Litopenaeus vannamei*.

## Figures and Tables

**Table 1:** Ingredients and proximate composition of experimental diets (% of dry matter).

Ingredients (%)	D1	D2	D3	D4	D5	D6	D7
Fish meal	20	15	15	15	15	15	15
Soybean meal	28	28	28	28	28	28	28
Peanut meal	12	12	12	12	12	12	12
Corn gluten meal	0	5	5	5	5	5	5
Wheat flour	23.85	23.25	23.248	23.246	23.244	23.242	23.24
Krill meal	2	2	2	2	2	2	2
Beer yeast	4	4	4	4	4	4	4
Fish oil	1.5	1.8	1.8	1.8	1.8	1.8	1.8
Soybean lecithin	1	1	1	1	1	1	1
Soy oil	1.5	1.5	1.5	1.5	1.5	1.5	1.5
Vitamin premix ^a^	1	1	1	1	1	1	1
Mineral premix ^b^	1	1	1	1	1	1	1
Choline	0.5	0.5	0.5	0.5	0.5	0.5	0.5
Ca(H_2_PO_4_)_2_	1.7	1.7	1.7	1.7	1.7	1.7	1.7
Lysine	0.29	0.53	0.53	0.53	0.53	0.53	0.53
Vitamin C	0.1	0.1	0.1	0.1	0.1	0.1	0.1
Methionine	0.24	0.25	0.25	0.25	0.25	0.25	0.25
Threonine	0.32	0.37	0.37	0.37	0.37	0.37	0.37
Sodium alginate	1	1	1	1	1	1	1
Coenzyme Q10 ^c^	0	0	0.002	0.004	0.006	0.008	0.01
Total	100	100	100	100	100	100	100
Proximate composition (%)							
Moisture	9.38	9.66	9.27	9.68	9.63	9.55	9.81
Crude protein	37.73	38.20	37.86	38.02	38.12	38.21	37.92
Crude lipid	6.96	7.24	7.02	7.12	7.32	7.18	7.07

Composition of vitamin premix (kg^−1^ of mixture): ^a^: vitamin A, 250,000 IU; riboflavin, 750 mg; pyridoxine HCL, 500 mg; cyanocobalamin, 1 mg; thiamin, 500 mg; menadione, 250 mg; folic acid, 125 mg; biotin, 10 mg; a-tocopherol, 3750 mg; myo-inositol, 2500 mg; calcium pantothenate, 1250 mg; nicotinic acid, 2000 mg; vitamin D3, 45,000 IU; vitamin C, 7000 mg. ^b^: Composition of mineral premix (kg^−1^ of mixture): Zn, 4000 mg; K, 22,500 mg; I, 200 mg; NaCl, 2.6 g; Cu, 500 mg; Co, 50 mg; FeSO_4_, 200 mg; Mg, 3000 mg; Se, 10 mg. ^c^: Coenzyme Q10 was provided by Zhejiang NHU Co., Ltd., Zhejiang, China.

**Table 2:** Primer information of quantitative real-time PCR.

Gene	Primer Sequence (5′ to 3′)	Genbank No.	Products Length
*β-actin* F	CTTGTGTGCGACAATGGCTC	XM_027364954.1	194
*β-actin* R	TCGATGGGGTACTTGAGGGT		
*MnSOD* F	TGTTGCACAAGCCATTGACG	XM_027381242.1	157
*MnSOD* R	ATCCTGGTTCTGGCAAGTGG		
*CAT* F	GCGACCAGAAACAACACACC	XM_027383071.1	166
*CAT* R	CTTGATGCCTTGGTCCGTCT		
*GPX* F	GGCACCAGGAGAACACTAC	XM_027368745.1	102
*GPX* R	CGACTTTGCCGAACATAAC		
*ProPO* F	TCCCGGACCGAGAAGATAGT	AY723296.1	105
*ProPO* R	TGTGGTATCATTCCCTGCGAG		
*LZM* F	CGATGATATCACGGAGGCCC	XM_027352857.1	111
*LZM* R	TTGCTGTTGTAAGCCACCCA		
*Toll* F	TTTCGGAGGATTGGAGTGCC	DQ923424.1	112
*Toll* R	GGTTTGTGAGGGAGTCCAGG		
*Myd88* F	CCTCAGCCCAGCTTCTAAACA	JX073566.1	110
*Myd88* R	CAGCCTGTTCTGCCAATCCT		
*TRAF6* F	GGAGGTTGCAGGACACATGA	HM581680.1	132
*TRAF6* R	TGTAGCTGCGTGGCTTGTAA		
*Pelle* F	CGTCAGTATTACGTCACGGC	KC346864	195
*Pelle* R	TGACTTCCAAGATGTGCGCT		
*Dorsal* F	CACGACCCATCAGAGTAGCC	XM_027382194.1	153
*Dorsal* R	AAACTGGAGGCTTCACAGCA		

**Table 3:** The effect of different coenzyme q10 levels on growth performance and feed utilization of *L. vannamei*.

Parameters	D1	D2	D3	D4	D5	D6	D7
IBW (g)	0.66 ± 0.01	0.67 ± 0.01	0.68 ± 0.00	0.66 ± 0.01	0.66 ± 0.00	0.68 ± 0.00	0.66 ± 0.01
FBW (g)	26.94 ± 0.78	24.71 ± 0.49	25.28 ± 0.47	25.71 ± 0.21	25.08 ± 1.17	26.08 ± 0.26	26.19 ± 0.77
WG (%)	3967.76 ± 120.48 _b_	3606.94 ± 62.50 _a_	3613.86 ± 70.97 _a_	3790.40 ± 25.70 _ab_	3708.63 ± 172.00 _ab_	3749.86 ± 42.72 _ab_	3899.68 ± 128.75 _ab_
SGR (%, day^−1^)	5.88 ± 0.05 _b_	5.73 ± 0.03 _a_	5.74 ± 0.03 _a_	5.81 ± 0.01 _ab_	5.77 ± 0.07 _ab_	5.79 ± 0.02 _ab_	5.85 ± 0.05 _ab_
FCR	1.46 ± 0.03	1.50 ± 0.03	1.48 ± 0.04	1.53 ± 0.09	1.56 ± 0.06	1.44 ± 0.03	1.51 ± 0.03
CF (g cm^−3^)	1.38 ± 0.03 _b_	1.28 ± 0.04 _a_	1.24 ± 0.03 _a_	1.23 ± 0.02 _a_	1.21 ± 0.02 _a_	1.22 ± 0.02 _a_	1.26 ± 0.01 _a_
Survival (%)	89.17 ± 5.51	89.17 ± 4.38	86.67 ± 4.91	88.33 ± 5.18	88.33 ± 3.97	90.00 ± 1.36	87.50 ± 1.60

Abbreviation: IBW, initial body weight; FBW, final body weight; WG, weight gain; SGR, specific growth rate; FCR, Feed conversion ration; CF, condition factor; SR, survival rate. Values are mean ± SEM of four replicates (n = 4); a,b, shown on the right of SEM indicated the significant difference among groups; different letters displayed on top indicated significant difference (*p* < 0.05); same letters mean no significant difference (*p* > 0.05).

**Table 4:** Whole-body proximate composition (%wet weight basis) of *L. vannamei fed* with different *level of coenzyme Q10*.

Parameter	D1	D2	D3	D4	D5	D6	D7
Moisture	72.77 ± 0.14 b	73.36 ± 0.37 ^bc^	74.43 ± 0.53 ^c^	71.06 ± 0.41 ^a^	72.77 ± 0.67 ^b^	74.06 ± 0.30 ^bc^	73.00 ± 0.75 ^bc^
Crude protein	19.23 ± 0.08	18.42 ± 0.08	18.46 ± 0.13	19.18 ± 0.42	18.05 ± 0.30	18.58 ± 0.30	19.10 ± 0.28
Crude lipid	1.75 ± 0.18	1.97 ± 0.05	1.90 ± 0.15	2.18 ± 0.12	2.02 ± 0.06	1.86 ± 0.11	2.01 ± 0.20

The results were expressed as mean ± standard error (Mean ± SEM); 4 replications in each group (n = 4); a,b,c, shown on the right of SEM indicate the significant differences among groups; different letters displayed on top indicated significant differences (*p* < 0.05); same letters mean no significant difference (*p* > 0.05).

## Data Availability

The data presented in this study are available in the article.

## References

[1] Fisheries Administration Bureau (2022). 2022 China Fishery Statistical Yearbook.

[2] FAO (2020). The State of World Fisheries and Aquaculture 2020. Sustainability in Action.

[3] Tacon A.G.J., Metian M. (2008). Global Overview on the Use of Fish Meal and Fish Oil in Industrially Compounded Aquafeeds: Trends and Future Prospects. Aquaculture.

[4] Olsen R.L., Hasan M.R. (2012). A Limited Supply of Fishmeal: Impact on Future Increases in Global Aquaculture Production. Trends Food Sci. Technol..

[5] Zhang C., Rahimnejad S., Wang Y.R., Lu K., Song K., Wang L., Mai K. (2018). Substituting Fish Meal with Soybean Meal in Diets for Japanese Seabass (*Lateolabrax japonicus*): Effects on Growth, Digestive Enzymes Activity, Gut Histology, and Expression of Gut Inflammatory and Transporter Genes. Aquaculture.

[6] Suárez J.A., Gaxiola G., Mendoza R., Cadavid S., Garcia G., Alanis G., Suárez A., Faillace J., Cuzon G. (2009). Substitution of Fish Meal with Plant Protein Sources and Energy Budget for White Shrimp *Litopenaeus vannamei* (Boone, 1931). Aquaculture.

[7] Erisman B.E., Bolser D.G., Ilich A., Frasier K.E., Glaspie C.N., Moreno P.T., Dell’Apa A., de Mutsert K., Yassin M.S., Nepal S. (2020). A Meta-Analytical Review of the Effects of Environmental and Ecological Drivers on the Abundance of Red Snapper (Lutjanus Campechanus) in the U.S. Gulf of Mexico. Rev. Fish Biol. Fish..

[8] Tacon A.G.J., Metian M., Hasan M.R. (2009). Feed Ingredients and Fertilizers for Farmed Aquatic Animals: Sources and Composition.

[9] Luthada-Raswiswi R., Mukaratirwa S., O’brien G. (2021). Animal Protein Sources as a Substitute for Fishmeal in Aquaculture Diets: A Systematic Review and Meta-Analysis. Appl. Sci..

[10] Molina-Poveda C., Lucas M., Jover M. (2013). Evaluation of the Potential of Andean Lupin Meal (*Lupinus mutabilis* Sweet) as an Alternative to Fish Meal in Juvenile *Litopenaeus vannamei* Diets. Aquaculture.

[11] Oujifard A., Seyfabadi J., Abedian Kenari A., Rezaei M. (2012). Growth and Apparent Digestibility of Nutrients, Fatty Acids and Amino Acids in Pacific White Shrimp, *Litopenaeus vannamei*, Fed Diets with Rice Protein Concentrate as Total and Partial Replacement of Fish Meal. Aquaculture.

[12] Yang Q., Tan B., Dong X., Chi S., Liu H. (2015). Effect of Replacing Fish Meal with Extruded Soybean Meal on Growth, Feed Utilization and Apparent Nutrient Digestibility of Juvenile White Shrimp (*Litopenaeus vannamei*). J. Ocean. Univ. China.

[13] Aya F.A., Cuvin-Aralar M.L., Coloso R.M. Potential of Cowpea (*Vigna Unguiculata* L.) Meal as an Alternative Protein Source in Diets for Giant Freshwater Prawn (Macrobrachium Rosenbergii, de Man 1879). Proceedings of the International Workshop on Resource Enhancement and Sustainable Aquaculture Practices in Southeast Asia 2014.

[14] Xie S., Niu J., Zhou W., Liu Y., Tian L. (2018). Developing a Low Fishmeal Diet for Juvenile Pacific White Shrimp, *Litopenaeus vannamei*, Using the Nutritional Value of FM as the Reference Profile. Aquac. Nutr..

[15] Habte-Tsion H.-M., Kolimadu G.D., Rossi W., Filer K., Kumar V. (2020). Effects of Schizochytrium and Micro-Minerals on Immune, Antioxidant, Inflammatory and Lipid-Metabolism Status of *Micropterus salmoides* Fed High- and Low-Fishmeal Diets. Sci. Rep..

[16] Bu X., Wang Y., Chen F., Tang B., Luo C., Wang Y., Ge X., Yang Y. (2018). An Evaluation of Replacing Fishmeal with Rapeseed Meal in the Diet of *Pseudobagrus ussuriensis*: Growth, Feed Utilization, Nonspecific Immunity, and Growth-related Gene Expression. J. World Aquac. Soc..

[17] Yigit M., Erdem M., Koshio S., Ergun S., Turker A., Karaali B. (2006). Substituting Fish Meal with Poultry by-Product Meal in Diets for Black Sea Turbot *Psetta maeotica*. Aquac. Nutr..

[18] Bureau D., Harris A., Bevan D., Simmons L., Azevedo P., Cho C. (2000). Feather Meals and Meat and Bone Meals from Different Origins as Protein Sources in Rainbow Trout (*Oncorhynchus mykiss*) Diets. Aquaculture.

[19] Tacon A.G.J. (1985). Utilisation of Conventional and Unconventional Protein Sources in Practical Fish Feeds. Nutrition and Feeding in Fish.

[20] Galkanda-Arachchige H.S., Wilson A.E., Davis D.A. (2020). Success of Fishmeal Replacement through Poultry by-Product Meal in Aquaculture Feed Formulations: A Meta-Analysis. Rev. Aquac..

[21] Novriadi R. (2017). A Meta-Analysis Approach toward Fish Meal Replacement with Fermented Soybean Meal: Effects on Fish Growth Performance and Feed Conversion Ratio. Asian Fish. Sci..

[22] Mugwanya M., Dawood M.A.O., Kimera F., Sewilam H. (2023). Replacement of Fish Meal with Fermented Plant Proteins in the Aquafeed Industry: A Systematic Review and Meta-Analysis. Rev. Aquac..

[23] Bautista-Teruel M.N., Fermin A.C., Koshio S.S. (2003). Diet Development and Evaluation for *Juvenile abalone*, Haliotis Asinina: Animal and Plant Protein Sources. Aquaculture.

[24] Dawood M.A.O., Koshio S. (2020). Application of Fermentation Strategy in Aquafeed for Sustainable Aquaculture. Rev. Aquac..

[25] McGoogan B.B., Gatlin D.M. (1997). Effects of Replacing Fish Meal with Soybean Meal in Diets for Red Drum *Sciaenops ocellatus* and Potential for Palatability Enhancement. J. World Aquac. Soc..

[26] Dias J., Gomes E.F., Kaushik S.J. (1997). Improvement of Feed Intake through Supplementation with an Attractant Mix in European Seabass Fed Plant-Protein Rich Diets. Aquat. Living Resour..

[27] Kader M.A., Koshio S., Ishikawa M., Yokoyama S., Bulbul M. (2010). Supplemental Effects of Some Crude Ingredients in Improving Nutritive Values of Low Fishmeal Diets for Red Sea Bream, *Pagrus major*. Aquaculture.

[28] Bae J., Hamidoghli A., Won S., Choi W., Lim S.-G., Kim K.-W., Lee B.-J., Hur S.-W., Bai S.C. (2020). Evaluation of Seven Different Functional Feed Additives in a Low Fish Meal Diet for Olive Flounder, *Paralichthys olivaceus*. Aquaculture.

[29] Yang X., He Y., Chi S., Tan B., Lin S., Dong X., Yang Q., Liu H., Zhang S. (2020). Supplementation with *Saccharomyces cerevisiae* Hydrolysate in a Complex Plant Protein, Low-Fishmeal Diet Improves Intestinal Morphology, Immune Function and *Vibrio harveyi* Disease Resistance in *Epinephelus coioides*. Aquaculture.

[30] Xie S.-W., Li Y.T., Zhou W.-W., Tian L.-X., Li Y.-M., Zeng S.-L., Liu Y.-J. (2017). Effect of γ-Aminobutyric Acid Supplementation on Growth Performance, Endocrine Hormone and Stress Tolerance of Juvenile Pacific white Shrimp, *Litopenaeus vannamei,* Fed Low Fishmeal Diet. Aquac. Nutr..

[31] Niu K.-M., Khosravi S., Kothari D., Lee W.-D., Lim J.-M., Lee B.-J., Kim K.-W., Lim S.-G., Lee S.-M., Kim S.-K. (2019). Effects of Dietary Multi-Strain Probiotics Supplementation in a Low Fishmeal Diet on Growth Performance, Nutrient Utilization, Proximate Composition, Immune Parameters, and Gut Microbiota of Juvenile Olive Flounder (*Paralichthys olivaceus*). Fish Shellfish. Immunol..

[32] Nepal P.R., Han H.-K., Choi H.-K. (2010). Enhancement of Solubility and Dissolution of Coenzyme Q10 Using Solid Dispersion Formulation. Int. J. Pharm..

[33] Greenberg S., Frishman W.H. (1990). Co-Enzyme Q10: A New Drug for Cardiovascular Disease. J. Clin. Pharmacol..

[34] Tran M.T., Mitchell T.M., Kennedy D.T., Giles J.T. (2001). Role of Coenzyme Q10 in Chronic Heart Failure, Angina, and Hypertension. Pharmacotherapy.

[35] Montano S.J., Grünler J., Nair D., Tekle M., Fernandes A.P., Hua X., Holmgren A., Brismar K., Ungerstedt J.S. (2015). Glutaredoxin Mediated Redox Effects of Coenzyme Q10 Treatment In Type 1 and Type 2 Diabetes Patients. BBA Clin..

[36] Sohmiya M., Tanaka M., Tak N.W., Yanagisawa M., Tanino Y., Suzuki Y., Okamoto K., Yamamoto Y. (2004). Redox Status of Plasma Coenzyme Q10 Indicates Elevated Systemic Oxidative Stress in Parkinson’s Disease. J. Neurol. Sci..

[37] Sugiyama S., Kitazawa M., Ozawa T., Suzuki K., Izawa Y. (1980). Anti-Oxidative Effect of Coenzyme Q10. Experientia.

[38] Park J., Park H.-H., Choi H., Kim Y.S., Yu H.-J., Lee K.-Y., Lee Y.J., Kim S.H., Koh S.-H. (2012). Coenzyme Q10 Protects Neural Stem Cells against Hypoxia by Enhancing Survival Signals. Brain Res..

[39] Amin M.M., Asaad G.F., Salam R.M.A., El-Abhar H.S., Arbid M.S. (2014). Novel CoQ10 Antidiabetic Mechanisms Underlie Its Positive Effect: Modulation of Insulin and Adiponectine Receptors, Tyrosine Kinase, PI3K, Glucose Transporters, sRAGE and Visfatin in Insulin Resistant/Diabetic Rats. PLoS ONE.

[40] Honda K., Kamisoyama H., Motoori T., Saneyasu T., Hasegawa S. (2010). Effect of Dietary Coenzyme Q10 on Cholesterol Metabolism in Growing Chickens. J. Poult. Sci..

[41] Varela-López A., Giampieri F., Battino M., Quiles J.L. (2016). Coenzyme Q and Its Role in the Dietary Therapy against Aging. Molecules.

[42] Henriksen J.E., Andersen C.B., Hother-Nielsen O., Vaag A., Mortensen S.A., Beck-Nielsen H. (1999). Impact of Ubiquinone (Coenzyme Q_10_) Treatment on Glycaemic Control, Insulin Requirement and Well-Being in Patients with Type 1 Diabetes Mellitus. Diabet. Med..

[43] Suksomboon N., Poolsup N., Juanak N. (2015). Effects of Coenzyme Q_10_ Supplementation on Metabolic Profile in Diabetes: A Systematic Review and Meta-Analysis. J. Clin. Pharm. Ther..

[44] Johansson P., Dahlström Ö., Dahlström U., Alehagen U. (2015). Improved Health-Related Quality of Life, and More Days Out of Hospital with Supplementation with Selenium and Coenzyme Q10 Combined. Results from a Double Blind, Placebo-Controlled Prospective Study. J. Nutr. Health Aging.

[45] Vetvicka V., Vetvickova J. (2018). Combination Therapy with Glucan and Coenzyme Q_10_in Murine Experimental Autoimmune Disease and Cancer. Anticancer. Res..

[46] Todorovic K., Jovanovic G., Todorovic A., Mitic A., Stojiljkovic N., Ilic S., Stojanovic N., Stojnev S. (2018). Effects of Coenzyme Q10 Encapsulated in Nanoliposomes on Wound Healing Processes after Tooth Extraction. J. Dent. Sci..

[47] Kessler R.C., Sonnega A., Bromet E., Hughes M., Nelson C.B. (1995). Posttraumatic Stress Disorder in the National Comorbidity Survey. Arch. Gen. Psychiatry.

[48] Shults C.W., Oakes D., Kieburtz K., Beal M.F., Haas R., Plumb S., Juncos J.L., Nutt J., Shoulson I., Carter J. (2002). Effects of Coenzyme Q10 in Early Parkinson Disease: Evidence of Slowing of the Functional Decline. Arch. Neurol..

[49] Fan L., Feng Y., Chen G.-C., Qin L.-Q., Fu C.-L., Chen L.-H. (2017). Effects of Coenzyme Q10 Supplementation on Inflammatory Markers: A Systematic Review and Meta-Analysis of Randomized Controlled Trials. Pharmacol. Res..

[50] De Barcelos I.P., Haas R.H. (2019). CoQ10 and Aging. Biology.

[51] Liao Z., Gong Y., Zhao W., He X., Wei D., Niu J. (2022). Comparison Effect of *Rhodobacter sphaeroides* Protein Replace Fishmeal on Growth Performance, Intestinal Morphology, Hepatic Antioxidant Capacity and Immune Gene Expression of *Litopenaeus vannamei* under Low Salt Stress. Aquaculture.

[52] Sánchez-Muros M.-J., Barroso F.G., Manzano-Agugliaro F. (2014). Insect Meal as Renewable Source of Food for Animal Feeding: A Review. J. Clean. Prod..

[53] Hardy R.W. (2010). Utilization of Plant Proteins in Fish Diets: Effects of Global Demand and Supplies of Fishmeal. Aquac. Res..

[54] Shiu Y.-L., Wong S.-L., Guei W.-C., Shin Y.-C., Liu C.-H. (2015). Increase in the Plant Protein Ratio in the Diet of White Shrimp, *Litopenaeus vannamei* (Boone), Using *Bacillus subtilis* E20-Fermented Soybean Meal as a Replacement. Aquac. Res..

[55] Liu X.-H., Ye J.-D., Wang K., Kong J.-H., Yang W., Zhou L. (2012). Partial Replacement of Fish Meal with Peanut Meal in Practical Diets for the Pacific White Shrimp, *Litopenaeus vannamei*. Aquac. Res..

[56] Molina-Poveda C., Cárdenas R., Jover M. (2017). Evaluation of Amaranth (*Amaranthus caudatus* L.) and Quinoa (*Chenopodium quinoa*) Protein Sources as Partial Substitutes for Fish Meal in *Litopenaeus vannamei* Grow-Out Diets. Aquac. Res..

[57] Wan M., Yin P., Fang W., Xie S., Chen S.J., Tian L.X., Niu J. (2018). The Effect of Replacement of Fishmeal by Concentrated Dephenolization Cottonseed Protein on the Growth, Body Composition, Haemolymph Indexes and Haematological Enzyme Activities of the Pacific White Shrimp (*Litopenaeus vannamei*). Aquac. Nutr..

[58] Hernández C., Lizárraga-Velázquez C.E., Contreras-Rojas D., Sánchez-Gutiérrez E.Y., Martínez-Montaño E., Ibarra-Castro L., Peña-Marín E.S. (2021). Fish Meal Replacement by Corn Gluten in Feeds for Juvenile Spotted Rose Snapper (*Lutjanus guttatus*): Effect on Growth Performance, Feed Efficiency, Hematological Parameters, Protease Activity, Body Composition, and Nutrient Digestibility. Aquaculture.

[59] Pratoomyot J., Bendiksen E., Bell J., Tocher D. (2010). Effects of Increasing Replacement of Dietary Fishmeal with Plant Protein Sources on Growth Performance and Body Lipid Composition of Atlantic Salmon (*Salmo salar* L.). Aquaculture.

[60] Regost C., Arzel J., Kaushik S. (1999). Partial or Total Replacement of Fish Meal by Corn Gluten Meal in Diet for Turbot (*Psetta maxima*). Aquaculture.

[61] Kumar V., Lee S., Cleveland B.M., Romano N., Lalgudi R.S., Benito M.R., McGraw B., Hardy R.W. (2020). Comparative Evaluation of Processed Soybean Meal (EnzoMealTM) vs. Regular Soybean Meal as a Fishmeal Replacement in Diets of Rainbow Trout (*Oncorhynchus mykiss*): Effects on Growth Performance and Growth-Related Genes. Aquaculture.

[62] Younis E.-S.M., Al-Quffail A.S., Al-Asgah N.A., Abdel-Warith A.-W.A., Al-Hafedh Y.S. (2018). Effect of Dietary Fish Meal Replacement by Red Algae, *Gracilaria arcuata*, on Growth Performance and Body Composition of Nile Tilapia *Oreochromis niloticus*. Saudi J. Biol. Sci..

[63] Sitjà-Bobadilla A., Peña-Llopis S., Gómez-Requeni P., Médale F., Kaushik S., Pérez-Sánchez J. (2005). Effect of Fish Meal Replacement by Plant Protein Sources on Non-Specific Defence Mechanisms and Oxidative Stress in Gilthead Sea Bream (*Sparus aurata*). Aquaculture.

[64] Xi L., Lu Q., Liu Y., Su J., Chen W., Gong Y., Han D., Yang Y., Zhang Z., Jin J. (2022). Effects of Fish Meal Replacement with Chlorella Meal on Growth Performance, Pigmentation, and Liver Health of Largemouth Bass (*Micropterus salmoides*). Anim. Nutr..

[65] Richard L., Surget A., Rigolet V., Kaushik S.J., Geurden I. (2011). Availability of Essential Amino Acids, Nutrient Utilisation and Growth in Juvenile Black Tiger Shrimp, *Penaeus monodon*, Following Fishmeal Replacement by Plant Protein. Aquaculture.

[66] Ye J., Liu X., Wang Z., Wang K. (2011). Effect of Partial Fish Meal Replacement by Soybean Meal on the Growth Performance and Biochemical Indices of Juvenile Japanese Flounder *Paralichthys olivaceus*. Aquac. Int..

[67] Feher J., Nemeth E., Nagy V., Lengyel G., Feher J. (2007). The Preventive Role of Coenzyme Q10 and Other Antioxidants in Injuries Caused by Oxidative Stress. Arch. Med. Sci..

[68] El Basuini M.F., Shahin S.A., Teiba I.I., Zaki M.A., El-Hais A.M., Sewilam H., Almeer R., Abdelkhalek N., Dawood M.A. (2021). The Influence of Dietary Coenzyme Q10 and Vitamin C on the Growth Rate, Immunity, Oxidative-Related Genes, and the Resistance against *Streptococcus agalactiae* of Nile Tilapia (*Oreochromis niloticus*). Aquaculture.

[69] El Basuini M.F., Teiba I.I., Zaki M.A., Alabssawy A.N., El-Hais A.M., Gabr A.A., Dawood M.A., Zaineldin A.I., Mzengereza K., Shadrack R.S. (2020). Assessing the Effectiveness of CoQ10 Dietary Supplementation on Growth Performance, Digestive Enzymes, Blood Health, Immune Response, and Oxidative-Related Genes Expression of Nile Tilapia (*Oreochromis niloticus*). Fish Shellfish. Immunol..

[70] Chang C.C., Ślesak I., Jordá L., Sotnikov A., Melzer M., Miszalski Z., Mullineaux P.M., Parker J.E., Karpińska B., Karpiński S. (2009). Arabidopsis Chloroplastic Glutathione Peroxidases Play a Role in Cross Talk between Photooxidative Stress and Immune Responses. Plant Physiol..

[71] Mahmoud H., Reda F., Alagawany M., Farag M. (2021). The Stress of Abamectin Toxicity Reduced Water Quality, Growth Performance, Immunity and Antioxidant Capacity of *Oreochromis niloticus* Fish: Modulatory Role of *Simmondsia chinensis* Extract as a Dietary Supplement. Aquaculture.

[72] Zhao W., Yao R., He X.-S., Liao Z.-H., Liu Y.-T., Gao B.-Y., Zhang C.-W., Niu J. (2022). Beneficial Contribution of the Microalga *Odontella aurita* to the Growth, Immune Response, Antioxidant Capacity, and Hepatic Health of Juvenile Golden Pompano (*Trachinotus ovatus*). Aquaculture.

[73] Wang Y., Li Z., Li J., Duan Y.-F., Niu J., Wang J., Huang Z., Lin H.-Z. (2015). Effects of Dietary Chlorogenic Acid on Growth Performance, Antioxidant Capacity of White Shrimp *Litopenaeus vannamei* under Normal Condition and Combined Stress of Low-Salinity and Nitrite. Fish Shellfish. Immunol..

[74] Pipe R., Porte C., Livingstone D. (1993). Antioxidant Enzymes Associated with the Blood Cells and Haemolymph of the Mussel *Mytilus edulis*. Fish Shellfish. Immunol..

[75] Garcia M.U., Foote C., van Es S., Devreotes P.N., Alexander S., Alexander H. (2000). Differential Developmental Expression and Cell Type Specificity of *Dictyostelium catalases* and Their Response to Oxidative Stress and UV-Light. Biochim. Biophys. Acta (BBA) Gene Struct. Expr..

[76] Margis R., Dunand C., Teixeira F.K., Margis-Pinheiro M. (2008). Glutathione Peroxidase Family—An Evolutionary Overview. FEBS J..

[77] Liu X.-L., Xi Q.-Y., Yang L., Li H.-Y., Jiang Q.-Y., Shu G., Wang S.-B., Gao P., Zhu X.-T., Zhang Y.-L. (2011). The Effect of Dietary *Panax ginseng* Polysaccharide Extract on the Immune Responses in White Shrimp, *Litopenaeus vannamei*. Fish Shellfish. Immunol..

[78] Zheng C., Zhao Q., Li E., Zhao D., Sun S. (2022). Role of hypoxia in the Behaviour, Physiology, Immunity and Response Mechanisms of Crustaceans: A Review. Rev. Aquac..

[79] Liao Z., Gong Y., Wang Z., Wang Y., Yao R., Chen M., Wei D., Zhao W., He X., Niu J. (2022). Effects of Dietary *Rhodobacter sphaeroides* Protein Substitution of Fishmeal and Coenzyme Q10 Supplementation on Growth Performance, Intestinal Microbiota and Stress Tolerance of *Litopenaeus vannamei* in Acute Low Salinity. Front. Mar. Sci..

[80] Bonakdar R.A., Guarneri E. (2005). Coenzyme Q10. Am. Fam. Physician.

[81] Mantle D., Heaton R.A., Hargreaves I.P. (2021). Coenzyme Q10 and Immune Function: An Overview. Antioxidants.

[82] Amparyup P., Charoensapsri W., Tassanakajon A. (2013). Prophenoloxidase System and Its Role in Shrimp Immune Responses against Major Pathogens. Fish Shellfish. Immunol..

[83] Paulsen S.M., Engstad R.E., Robertsen B. (2001). Enhanced Lysozyme Production in Atlantic Salmon (*Salmo salar* L.) Macrophages Treated with Yeast β-Glucan and Bacterial Lipopolysaccharide. Fish Shellfish. Immunol..

[84] Littarru G.P. (1993). Biomedical and Clinical Aspects of Coenzyme Q. Clin. Investig..

[85] Crane F.L., Sun I.L., Sun E.E. (1993). The Essential Functions of Coenzyme Q. Clin. Investig..

[86] Yang M., Sun S., Fu H., Qiao H., Zhang W., Gong Y., Jiang S., Xiong Y., Xu L., Zhao C. (2019). Hypoxia and Reoxygenation on Antioxidant Enzyme Activities and Histological Structure of *Macrobrachium nipponense*. J. Fish. Sci. China.

[87] Sun S., Xuan F., Fu H., Zhu J., Ge X., Gu Z. (2015). Transciptomic and Histological Analysis of Hepatopancreas, Muscle and Gill Tissues of Oriental River Prawn (*Macrobrachium nipponense*) in Response to Chronic Hypoxia. BMC Genom..

[88] Duan Y., Zhang J., Dong H., Wang Y., Liu Q., Li H. (2016). Effect of Desiccation and Resubmersion on the Oxidative Stress Response of the Kuruma Shrimp *Marsupenaeus japonicus*. Fish Shellfish. Immunol..

[89] Sun S., Xuan F., Ge X., Fu H., Zhu J., Zhang S. (2014). Identification of Differentially Expressed Genes in Hepatopancreas of Oriental River Prawn, *Macrobrachium nipponense* Exposed to Environmental Hypoxia. Gene.

[90] Zenteno-Savín T., Saldierna R., Ahuejote-Sandoval M. (2006). Superoxide Radical Production in Response to Environmental Hypoxia in Cultured Shrimp. Comp. Biochem. Physiol. Part. C Toxicol. Pharmacol..

[91] Yang S., Fan B., Chen X., Meng Z. (2021). Supplementation of the Freezing Medium with Coenzyme Q10 Attenuates Oxidative Stress and Improves Function of Frozen-Thawed Giant Grouper (*Epinephelus lanceolatus*) Spermatozoa. Theriogenology.

[92] Chenga W., Liub C.H., Hsuc J.P., Chend J.C. (2002). Effect of Hypoxia on the Immune Response of Giant Freshwater Prawn *Macrobrachium rosenbergii* and Its Susceptibility to Pathogen *Enterococcus*. Fish Shellfish. Immunol..

[93] Le Moullac G., Soyez C., Saulnier D., Ansquer D., Avarre J.C., Levy P. (1998). Effect of Hypoxic Stress on the Immune Response and the Resistance to Vibriosis of the Shrimp *Penaeus stylirostris*. Fish Shellfish. Immunol..

[94] Li C., Wang S., He J. (2019). The Two NF-ΚB Pathways Regulating Bacterial and WSSV Infection of Shrimp. Front. Immunol..

[95] Valanne S., Wang J.-H., Rämet M. (2011). The *Drosophila* Toll Signaling Pathway. J. Immunol..

[96] Huang X.-D., Yin Z.-X., Jia X.-T., Liang J.-P., Ai H.-S., Yang L.-S., Liu X., Wang P.-H., Li S.-D., Weng S.-P. (2010). Identification and Functional Study of a Shrimp *Dorsal homologue*. Dev. Comp. Immunol..

[97] Huang X., Wang W., Ren Q. (2016). Dorsal Transcription Factor Is Involved in Regulating Expression of Crustin Genes during White Spot Syndrome Virus Infection. Dev. Comp. Immunol..

